# New species of Rissoidae (Mollusca, Gastropoda) from the Archipelago of the Azores (northeast Atlantic) with an updated regional checklist for the family

**DOI:** 10.3897/zookeys.480.8599

**Published:** 2015-02-02

**Authors:** Ricardo Cordeiro, Sérgio P. Ávila

**Affiliations:** 1 CIBIO, Centro de Investigação em Biodiversidade e Recursos Genéticos, InBIO Laboratório Associado, Pólo dos Açores, Universidade dos Açores, Campus de Ponta Delgada, Apartado 1422, 9501–801 Ponta Delgada, Açores, Portugal; 2 Departamento de Biologia, Universidade dos Açores, Campus de Ponta Delgada, Apartado 1422, 9501–801 Ponta Delgada, Açores, Portugal; 3 Faculdade de Ciências da Universidade do Porto, Rua Campo Alegre 1021/1055, 4169–007 Porto, Portugal

**Keywords:** Taxonomy, Caenogastropoda, Rissooidea, *Setia*, *Manzonia*, Eastern Atlantic

## Abstract

Four new species of shallow-water marine gastropods belonging to the family Rissoidae are described from the Archipelago of the Azores: *Setia alexandrae* **sp. n.**, *Setia ermelindoi* **sp. n.**, *Setia netoae* **sp. n.**, and *Manzonia martinsi* **sp. n.** These novelties increase the regional rissoid fauna to 39 species, of which 29 live in shallow-water habitats. A list of the species of Rissoidae from the Azores is presented based on data from the literature and new material examined.

## Introduction

Molluscs are among the best known marine invertebrates in the Archipelago of the Azores. Several publications have addressed this subject in recent decades, resulting in a consistent increase of the mollusc richness for the Azores based on the description and record of species (Amati 1987, Gofas 1989, 1990, Hoenselaar and Goud 1998, Aartsen 2008, Malaquias et al. 2009, Martins et al. 2009, Ávila et al. 2011, Malaquias et al. 2011, Pedro et al. 2011, Ávila and Sigwart 2013, Cordeiro et al. 2013).

The members of the family Rissoidae Gray, 1847 are amongst the most conspicuous, abundant and diverse gastropods inhabiting the continental shelf and upper bathyal region in the Mediterranean Sea and along the Atlantic coasts of Europe (for a review see Ávila et al. 2012). Such gastropods are also very abundant in the northeast Atlantic seamounts (Ávila and Malaquias 2003, Ávila et al. 2004, 2007, Gofas 2007) as well as in the Archipelago of the Azores (Dautzenberg 1889, Aartsen 1982, Amati 1987, Moolenbeek and Faber 1987, Gofas 1989, 1990, Bouchet and Warén 1993, Hoenselaar and Goud 1998, Ávila 2000, Costa and Ávila 2001, Martins et al. 2009), Madeira, Porto Santo and Desertas Islands (Manzoni 1868a, 1868b, Watson 1873, Nobre 1937, Moolenbeek and Faber 1987, Moolenbeek and Hoenselaar 1989, 1998, Hoenselaar and Goud 1998, Segers et al. 2009), Selvagens Islands (Albuquerque et al. 2009), Canary Islands (Rolán 1987, Verduin 1988, Moolenbeek and Hoenselaar 1989, 1992, 1998, Hernández-Otero and García 2003) and Archipelago of Cape Verde (Rolán 1987, Moolenbeek and Rolán 1988, Templado and Rolán 1993, Rolán and Rubio 1999, Rolán and Luque 2000, Rolán 2005, Rolán and Oliveira 2008).

Ávila (2000, 2005) detailed the shallow-water (from the intertidal down to 50 m depth) rissoids of the Azores, identifying a total of 9 genera and 24 species. Ávila et al. (2002, 2010) summarized the palaeontological record for the family in the area. The bathymetrical zonation for the most common rissoid species was described by Ávila (2003) and Ávila et al. (2005).

The genus *Setia* H. & A. Adams, 1852 is composed of minute gastropods with ovate to ovate-conic shells and convex whorls. The dome-shaped protoconch, having about 1.25 to 1.5 whorls, is smooth or possesses spiral rows of minute gemmae. The teleoconch is smooth or sculptured with weak to moderate spiral threads and axial growth lines. The aperture has a simple peristome.

There are 29 species of *Setia* in the Atlantic and the Mediterranean Sea (Ávila et al. 2012). With the exception of *Setia triangularis* (Watson, 1886) reported for the Caribbean and Ascension Island, all are shallow-water species. No species of this genus are reported for the Lusitanian seamounts (Gofas 2007). The Mediterranean Sea contains the largest number of species (18 species; 10 endemic), followed by the Canary Islands (7 species; 1 endemic), mainland Portugal (6 species), Archipelago of Madeira (4 species; 1 endemic), and the Azores (3 species; 2 endemic) (Ávila et al. 2012).

The genus *Manzonia* Brusina, 1870 is composed of minute gastropods with ovate-conic shells having robust axial sculpture formed by strongly curved to sinuous opisthocline ribs. The protoconch is multispiral (with netted microsculpture) or paucispiral (with gemmate or smooth spiral keels). The distinctive teleoconch microsculpture consists of a pitted surface on the flat spiral cords and very fine spiral threads in the interspaces between primary cords. The aperture is oval and the peristome is duplicated (Ponder 1985, Moolenbeek and Faber 1987).

A total of 24 species of *Manzonia* is present in the Atlantic and the Mediterranean Sea (Ávila et al. 2012). Of these, 6 are deep-water and 18 are shallow-water species. The deep-water species are restricted to the Lusitanian seamounts (Gofas 2007) and the West African coast (Gofas 2010). Twenty three of the 24 species are distributed in both the eastern Atlantic and the Mediterranean Sea. The remaining species, *Manzonia epima* (Dall & Simpson, 1901), is restricted to the western Atlantic. The Canary Islands (11 species; 3 endemic), Selvagens Islands (8 species; 1 endemic) and Archipelago of Madeira (7 species; 2 endemic) contain the largest numbers of species. The Archipelago of the Azores contains a single, endemic species (Ávila et al. 2012).

The present study describes three new species of *Setia* and a new species of *Manzonia* from the Archipelago of the Azores. A list of the species of Rissoidae from the Azores is presented based on data from the literature and new material examined.

## Materials and methods

The specimens used in the present study were obtained from samples collected by about 850 dives and dredges in all islands of the Azores between the years 1967 and 2010. A total of 1,060 lots from the Mollusc Reference Collection of the Department of Biology of the University of the Azores (DBUA) were examined. During the course of examining the material, we found a total of 39,532 specimens of Rissoidae. All specimens were examined under stereomicroscope. The specimens in perfect conditions were selected as type material for the new species and measured with precision of 0.01 mm using a stereomicroscope with a digital camera coupled to a computer.

Living animals were photographed for observation of colour patterns. Shells were sonicated, coated with Au-Pd and then photographed with a Scanning Electron Microscope (SEM) for the study of the protoconch and teleoconch.

Terminology adopted for shell descriptions follows Ponder (1985). A special focus was placed on protoconch and microsculpture as taxonomic characters at the species level. The suprageneric classification of Gastropoda is based on Bouchet and Rocroi (2005).

The material analysed in this study is deposited in the Mollusc Reference Collection of the Department of Biology of the University of the Azores (DBUA), Portugal. Abbreviations used: sh. – shell; spc. – specimens alive when collected.

## Systematics

### Class GASTROPODA Cuvier, 1795 Subclass CAENOGASTROPODA Cox, 1960 Order LITTORINIMORPHA Golikov & Starobogatov, 1975 Superfamily RISSOOIDEA Gray, 1847 Family RISSOIDAE Gray, 1847

#### Genus Setia

Taxon classificationAnimaliaLittorinimorphaRissoidae

H. & A. Adams, 1852

##### Type species.

*Rissoa pulcherrima* Jeffreys, 1848; subsequent designation Kobelt 1878: 128.

#### Setia alexandrae

Taxon classificationAnimaliaLittorinimorphaRissoidae

Ávila & Cordeiro sp. n.

http://zoobank.org/19AAF0E8-8392-49B2-BCB8-E50C96F266E8

Figure 1

Setia sp.: Ávila et al. (1998: 496) – DBUA 449, 478, 496, 662.
Setia sp.: Ávila et al. (2000: 147) – DBUA 787.
Setia sp.: Costa and Ávila (2001: 126) – DBUA 898 (as ATA 1).
Setia sp.: Ávila (2003: 32) – DBUA 704/A, 787.

##### Type material.

Holotype, DBUA 1051 (spc., 1.17 × 0.70 mm); paratype 1, DBUA 1070 (spc., 0.99 × 0.65 mm); paratype 2, DBUA 1071 (spc., 1.09 × 0.73 mm); paratype 3, DBUA 1072 (spc., 1.08 × 0.66 mm); paratype 4, DBUA 1073 (spc., 1.04 × 0.65 mm), Graciosa Island (Santa Cruz, intertidal, 01/08/2010); paratype 5, DBUA 1074 (sh., 0.95 × 0.65 mm); paratype 6, DBUA 1075 (sh., 1.02 × 0.61 mm); paratype 7, DBUA 1076 (sh., 1.07 × 0.68 mm), Pico Island (Lajes do Pico, intertidal, 07/1989); paratype 8, DBUA 1077 (spc., 0.91 × 0.62 mm); paratype 9, DBUA 1078 (spc., 1.10 × 0.65 mm), São Miguel Island (Caloura, 5–15 m depth, 21/05/1999).

##### Type locality.

Santa Cruz, Graciosa Island, Azores.

##### Additional material examined.

Graciosa Island: DBUA 35 (Fonte da Areia, intertidal, 3 spc., 10/06/1988); DBUA 37 (Porto Afonso, intertidal, 14 spc., 06/1988); DBUA 40 (Santa Cruz, intertidal, 1 sh., 06/1988); DBUA 48 (Baía da Folga, infralittoral, 1 sh., 06/1988); DBUA 50 (Baía da Folga, 8 m depth, 1 spc., 10/06/1988). Pico Island: DBUA 449 (Lajes do Pico, intertidal, 5 spc., 07/1989); DBUA 468 (Lajes do Pico, intertidal, 11 spc., 07/1989); DBUA 471 (Lajes do Pico, intertidal, 1 spc., 07/1989); DBUA 478 (Lajes do Pico, intertidal, 7 spc., 07/1989), DBUA 496 (Lajes do Pico, intertidal, 31 spc., 28/06/1991); and DBUA 662 (Lajes do Pico, 0–3 m depth, 1 sh., 19/08/1995). São Miguel Island: DBUA 704/A (São Vicente, 12 m depth, 1 spc., 18/07/1996); DBUA 787 (São Vicente, 15.1 m depth, 2 spc., 11/07/1997), DBUA 898 (Atalhada, 11.2 m depth, 4 spc., 10/10/1996), DBUA 901 (Ilhéu de Vila Franca do Campo, 15 m depth, 1 spc., 15/07/1996), DBUA 920 (Caloura, 10 m depth, 1 spc., 12/07/1997), DBUA 963 (Porto de Vila Franca do Campo, 6 m depth, 2 spc., 22/07/1997). Formigas Islets: DBUA 336 (intertidal, 2 spc., 07/1990); and DBUA 355 (15 m depth, 22 spc., 03/07/1991). Santa Maria Island: DBUA 1018 (Ilhéu da Vila, 17 m depth, 6 spc., 5 sh., 26/08/2004); and DBUA 1019 (Ilhéu da Vila, 17 m depth, 2 spc., 26/08/2004).

##### Etymology.

Named after Alexandra Castela, the wife of Sérgio Ávila.

##### Description.

**Figure 1.**
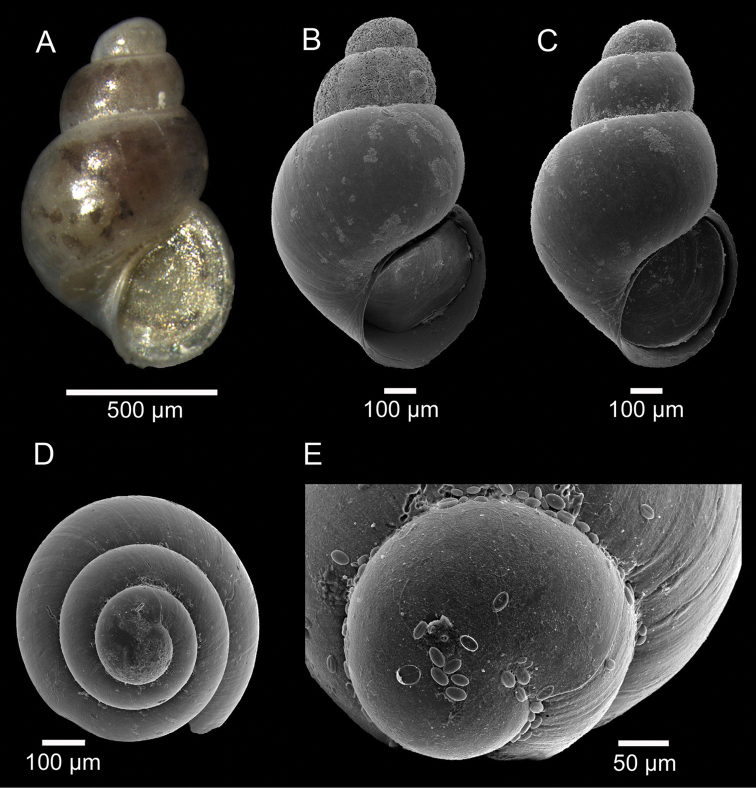
*Setia alexandrae* sp. n. **A** Holotype (1.17 × 0.70 mm), DBUA 1051 (shell) **B** Paratype 3 (1.08 × 0.66 mm), DBUA 1072 (shell) **C** Paratype 9 (1.10 × 0.65 mm), DBUA 1078 (shell) **D** Paratype 8, DBUA 1077 (shell, apical view) **E** Paratype 2, DBUA 1071 (protoconch).

Shell fragile, minute, translucent, oval-conical, up to 1.2 × 0.7 mm (Fig. 1A). Protoconch smooth, dome-shaped (typical of the genus), whorls 1.25, diameter 280 µm, separated from the teleoconch by a clearly visible line (Fig. 1D and E). Teleoconch with 2.5 to 3 inflated, rounded, strongly convex whorls; whorls with regular contour and conspicuous increase in width (Fig. 1B–D). Spire moderately high. Sculpture absent, except for very fine, inconspicuous growth lines (Fig. 1B–D). Suture deep, constricted (Fig. 1B–D). Last whorl large, globose, 70–75% of shell length (Fig. 1B and C). Base large, rounded. Aperture oval, oblique with continuous peristome, adapical angle somewhat acute (Fig. 1B and C). Parietal region thin, very slightly convex (Fig. 1B and C). Outer lip with very thin edge (Fig. 1B and C). Inner lip thin, very slightly recurved over umbilicus (Fig. 1B and C). Umbilicus a very narrow fissure (Fig. 1B and C). Animal light-yellow with dark-brown blotches visible at transparency (Fig. 1A). Operculum simple, thin, nucleus eccentric, yellowish at transparency (Fig. 1A and B).

##### Habitat.

On rocky shores covered by algae, from the intertidal down to 20 m depth.

##### Geographical distribution.

Pico, Graciosa, São Miguel and Santa Maria Islands, and Formigas Islets. Probably endemic to the Azores.

##### Remarks.

*Setia alexandrae* sp. n. is distinguished from the other Azorean congeners by its smooth, transparent to translucent shell, and yellowish animal having light to dark-brown blotches over the entire body. *Setia alexandrae* sp. n. lacks any colour decoration/pattern on the shell. Colour is a distinctive feature of most of the known *Setia* (e.g., variably coloured flames, spots or vertical lines, sometimes interrupted medially), which are so abundant on the Azorean *Setia subvaricosa* Gofas, 1990, *Setia amabilis* (Locard, 1886), *Setia ambigua* (Brugnone, 1873) and *Setia scillae* (Aradas & Benoit, 1876). *Setia lacourti* (Verduin, 1984) is similar in size (up to 1.2 mm in length) and also lacks any coloured pattern on the shell; this species was reported from the Azores by Segers (2002: 89) as Setia cf. lacourti. *Setia alexandrae* sp. n. clearly differs from *Setia lacourti* in shell shape (more elongated on the first, flattened on the latter). *Setia ambigua* was also reported from the Azores (Hoenselaar and Goud in litt. 2002, Ávila et al. 2012), from Terceira Island (Praia da Vitória, 38°43'N, 27°04'W, sandy beach). *Setia alexandrae* sp. n. differs from *Setia ambigua* by being about 50% smaller and by lacking any coloured decoration on the shell. A very similar specimen to *Setia alexandrae* sp. n. was recently found in the Pleistocene record of Santa Maria Island, Azores (Prainha outcrop, bed B1 of Ávila et al. (2009a); latitude 36°57'3.05"N, longitude 25°6'44.20"W). This well-preserved juvenile shell is herein reported as Setia cf. alexandrae DBUA-F 137/151-4 (0.61 × 0.40 mm). The protoconch and teleoconch shape and sculpture are identical to *Setia alexandrae* sp. n., as is the shape of the aperture. However, as it is a juvenile specimen we prefer to treat it as Setia cf. alexandrae until adult material is examined.

#### Setia ermelindoi

Taxon classificationAnimaliaLittorinimorphaRissoidae

Ávila & Cordeiro sp. n.

http://zoobank.org/E6DF8087-FD10-4A01-8324-8A24E56C00C3

Figure 2

Setia cf. lacourti (Verduin, 1984): Segers (2002: 89).

##### Type material.

Holotype, DBUA 1058 (sh., 1.07 × 0.76 mm), São Miguel Island (Caloura, 5–15 m depth, 21/05/1999); paratype 1, DBUA 1079 (spc., 1.08 × 0.80 mm), Pico Island (Lajes do Pico, 1–2 m depth, 24/06/1991); paratype 2, DBUA 1080 (spc., 0.74 × 0.59 mm); paratype 3, DBUA 1081 (spc., 0.92 × 0.76 mm), Pico Island (Lajes do Pico, intertidal, 24/06/1991); paratype 4, DBUA 1082 (spc., 0.94 × 0.70 mm), Flores Island (Santa Cruz, intertidal, 09/07/1989); paratype 5, DBUA 1083 (spc., 0.79 × 0.63 mm); paratype 6, DBUA 1084 (spc., 0.94 × 0.66 mm); paratype 7, DBUA 1085 (spc., 0.78 × 0.62 mm), Flores Island (Santa Cruz, intertidal, 08/08/2010).

##### Type locality.

Caloura, São Miguel Island, Azores.

##### Additional material examined.

Pico Island: DBUA 467 (Lajes do Pico, intertidal, 1 sh., 07/1989). São Miguel Island: DBUA 137 (Ilhéu de Vila Franca do Campo, intertidal, 2 spc., 07/1988); DBUA 689 (São Vicente, 22 m depth, 1 sh., 15/07/1996); DBUA 899 (Faial da Terra, 8.3 m depth, 2 spc., 10/10/1996); DBUA 957 (Pesqueiro, 5.6 m depth, 1 spc., 19/07/1997).

##### Etymology.

Named after Ermelindo Ávila, a writer and historian from Pico Island.

##### Description.

**Figure 2.**
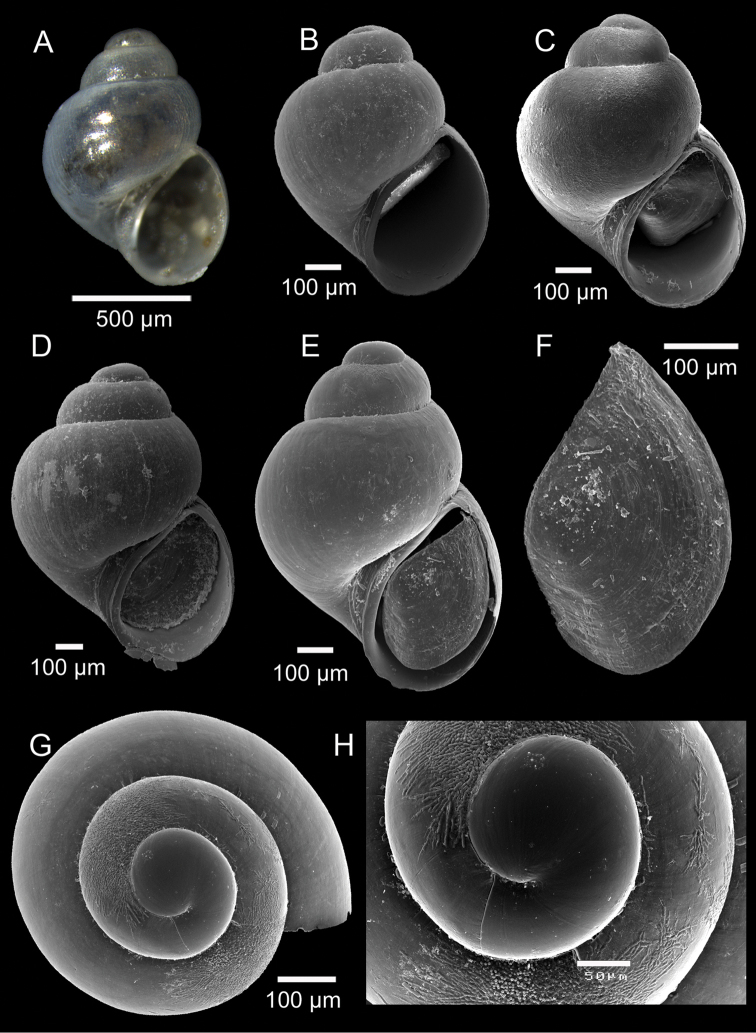
*Setia ermelindoi* sp. n. **A** Holotype (1.07 × 0.76 mm), DBUA 1058 **B** Paratype 2 (0.74 × 0.59 mm), DBUA 1080 (shell) **C** Paratype 5 (0.79 × 0.63 mm), DBUA 1083 (shell) **D** Paratype 1 (1.08 × 0.80 mm), DBUA 1079 (shell) **E** Paratype 6 (0.94 × 0.66 mm), DBUA 1084 (shell) **F** Paratype 6, DBUA 1084 (operculum) **G** Paratype 7, DBUA 1085 (apical view) **H** Paratype 7, DBUA 1085 (protoconch).

Shell minute, translucent, globose, up to 1.1 × 0.8 mm (Fig. 2A). Protoconch paucispiral, whorls 1.25, diameter 225–235 µm, smooth, with no visible sculpture, except for a few faintly developed axial growth lines, separated from the teleoconch by a clearly visible line (Fig. 1G and H). Teleoconch with 1.75 to 2 inflated, rounded, strongly convex whorls sculptured by faint axial lines (no spiral sculpture present); whorls with regular contour and conspicuous increase in width (Fig. 2B–E). Spire short. Suture deep, constricted (Fig. 2B–E). Last whorl very large, globose, 75–80% of shell length (Fig. 2B–E). Base large, rounded. Aperture oval and oblique with faint posterior angulation (Fig. 2B–E). Parietal region moderately thickened, rather straight to very slightly convex (Fig. 2B–E). Outer lip thin, smooth inner surface (Fig. 2B–D). Inner lip thin, slightly reflected over umbilicus (Fig. 2B–E). Umbilicus a moderately enlarged fissure (Fig. 2B–E). One axial threads always present and running abapically nearby the umbilicus (Fig. 2D). Animal black (Fig. 2A). Foot whitish. Operculum simple, thin, nucleus eccentric, translucent (Fig. 2E and F).

##### Habitat.

On rocky shores covered by algae, from the intertidal down to 25 m depth.

##### Geographical distribution.

Flores, Pico and São Miguel Islands. Probably endemic to the Azores.

##### Remarks.

This species differs from most of the known *Setia* species by its globose shell. *Setia lacourti* (Verduin, 1984) and *Setia valvatoides* (Milaschewitsch, 1909) also have globose shells, but *Setia ermelindoi* sp. n. is easily separated from these species by the aperture shape, which is oval in *Setia ermelindoi* sp. n. and rounded in *Setia lacourti* and *Setia valvatoides*. *Setia ermelindoi* sp. n. is also distinguished by the axial thread running abapically near the umbilicus. The shell shape of *Setia ermelindoi* sp. n. resembles that of some *Rissoella* species (e.g., *Rissoella contrerasi* Rolán & Hernández, 2004 and *Rissoella inflata* (Monterosato, 1880)). However, the animal lacks the typical two pairs of head tentacles found in all *Rissoella*; instead, it has only one pair typical of Rissoidae. Moreover, *Setia ermelindoi* sp. n. does not have the pigmented hypobranchial gland (observable through the transparent shell) of several *Rissoella* (a species-diagnostic character, cf. Rolán and Hernández 2003; e.g., *Rissoella contrerasi*, *Rissoella luteonigra* Rolán & Rubio, 2001, *Rissoella trigoi* Rolán & Hernández, 2004, *Rissoella caribaea* Rehder, 1943, and *Rissoella ornata* Simone, 1995).

#### Setia netoae

Taxon classificationAnimaliaLittorinimorphaRissoidae

Ávila & Cordeiro sp. n.

http://zoobank.org/CFE8729E-CB9E-466B-A352-42E806D18A73

Figure 3

##### Type material.

Holotype, DBUA 745 (1 spc., 1.57 × 0.90 mm); paratype 1, DBUA 1086 (1 spc., 1.55 × 0.89 mm), São Miguel Island (Baía de Rosto do Cão, low intertidal, 07/1990); paratype 2, DBUA 1087 (1 spc., 1.15 × 0.70 mm); paratype 3, DBUA 1088 (1 spc., 1.17 × 0.71 mm), São Miguel Island (Baía de Belém, São Roque, 8.6 m depth, 04/07/1990); paratype 4, DBUA 1089 (spc., 1.26 × 0.74 mm); paratype 5, DBUA 1090 (spc., 1.12 × 0.68 mm), paratype 6, DBUA 1091 (spc., 1.36 × 0.79 mm), Graciosa Island (Baía da Folga, 8 m depth, 10/06/1988); paratype 7, DBUA 264 (1 sh., 2.09 × 1.07 mm), Flores Island (Lajes das Flores, 6–10 m depth, 27/10/1990).

##### Type locality.

Baía de Rosto do Cão, São Miguel Island, Azores.

##### Etymology.

Named after Ana Neto, an Azorean marine phycologist from the University of the Azores.

##### Description.

**Figure 3.**
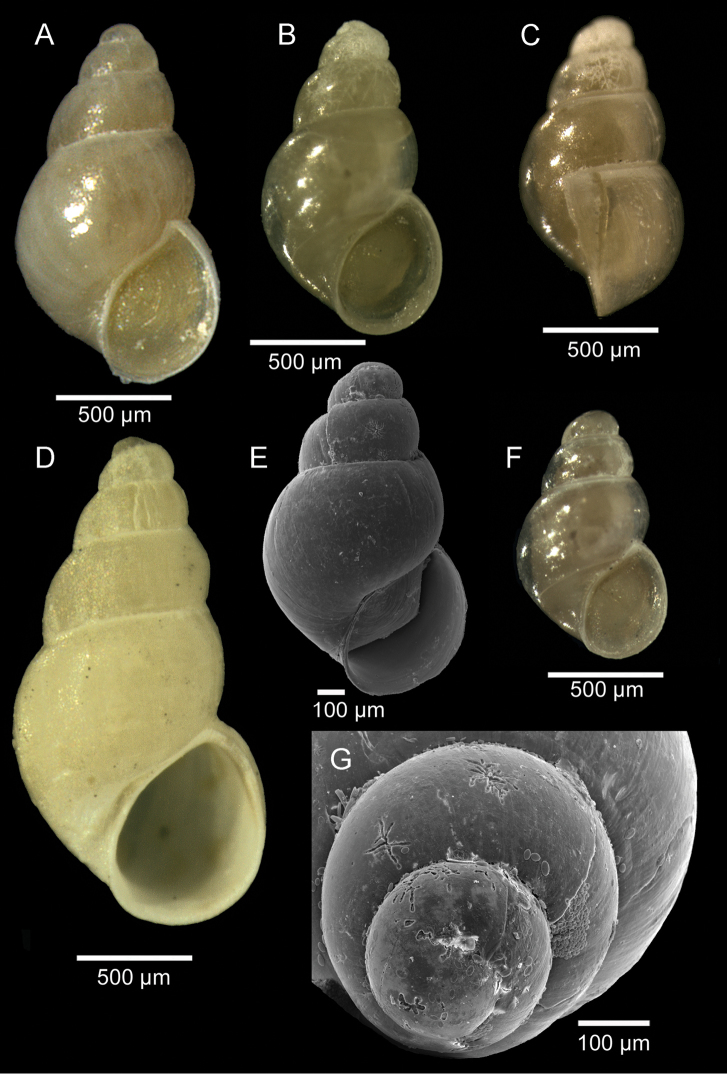
*Setia netoae* sp. n. **A** Holotype (1.57 × 0.90 mm mm), DBUA 745 (shell) **B** Paratype 6 (1.36 × 0.79 mm mm), DBUA 1091 (shell) **C** Paratype 6, DBUA 1091 (shell, lateral view) **D** Paratype 7 (2.09 × 1.07 mm), DBUA 264 (shell) **E** Paratype 1 (1.55 × 0.89 mm), DBUA 1086 (shell) **F** Paratype 5 (1.12 × 0.68 mm), DBUA 1090 (shell) **G** Paratype 1, DBUA 1086 (protoconch, apical view).

Shell minute, cream to translucent in colour, oval-high conical, up to 2.1 × 1.1 mm (Fig. 3A–D and F). Protoconch dome-shaped (typical of the genus), smooth, whorls 1.25, diameter 250 µm, separated from the teleoconch by a clearly visible line (Fig. 3G). Teleoconch with 3.5 to 4 inflated, rounded, strongly convex whorls sculptured with weak axial growth lines; whorls with regular contour and conspicuous increase in width (Fig. 3A–F). Spire moderately high. Suture deep, constricted (Fig. 3E). Last whorl large, globose, 60% of shell length (Fig. 3A–F). Base large, rounded (Fig. 3E). Aperture oval, oblique with continuous and simple peristome (smooth within), and posterior angulation (Fig. 3E). Parietal region thin, rather straight (Fig. 3E). Outer and inner lip with a thin edge (not thickened) (Fig. 3E). Umbilicus a very narrow fissure (Fig. 3E). Animal bright-yellow, with very distinctive single dark-brown patch to inner side of head, readily visible at transparency (Fig. 3A–C and F). Operculum simple, thin, nucleus eccentric, yellowish at transparency (Fig. 3A, B and F).

##### Habitat.

On rocky shores covered by algae, from the intertidal down to 10 m depth.

##### Geographical distribution.

Flores, Graciosa and São Miguel Islands. Probably endemic to the Azores.

##### Remarks.

This species appears to be uncommon on the Azorean shores. It differs from *Setia subvaricosa* by lacking the vertical reddish flames on the shell that characterizes this species. It further differs from *Setia subvaricosa* by the thinner outer lip of the aperture and by the absence of spiral threads on the protoconch. *Setia netoae* sp. n. differs from *Setia alexandrae* sp. n. in having a more slender shell, 4 instead of 3 whorls on the teleoconch, by the distinctive colour pattern of the animal, and by the proportions of the last whorl/total length of the shell, which are 60% versus 70–75%, respectively. *Setia netoae* sp. n. differs from other Atlantic congeners (cf. Ávila et al. 2012; and present study) by lacking the usual colourful patterns, such as wavy flames, blotches and lines, observed in the shells of this genus. It also has a more elongate shell than most of the known *Setia*. The shell resembles that of similar sized *Setia antipolitana* (van der Linden & Wagner, 1987), but the latter has vertical lines interrupted medially that are not present in *Setia netoae* sp. n.

#### Genus Manzonia

Taxon classificationAnimaliaLittorinimorphaRissoidae

Brusina, 1870

##### Type species.

*Turbo costatus* J. Adams, 1798, by original designation (=*Turbo crassus* Kanmacher, 1798; non *Turbo costatus* von Salis Marschlins, 1793).

#### Manzonia martinsi

Taxon classificationAnimaliaLittorinimorphaRissoidae

Ávila & Cordeiro sp. n.

http://zoobank.org/78D90675-432F-455F-B042-35896D37CCD2

Figure 4

##### Type material.

Holotype, DBUA 788 (sh., 1.75 × 1.13 mm); paratype 1, DBUA 1092 (sh., 1.88 × 1.20 mm); paratype 2, DBUA 1093 (sh., 1.76 × 1.14 mm); paratype 3, DBUA 1094 (sh., 1.72 × 1.10 mm); paratype 4, DBUA 1095 (sh., 1.76 × 1.06 mm), São Miguel Island (São Vicente Ferreira, 4.7 m depth, 16/07/1997).

##### Type locality.

São Vicente Ferreira, São Miguel Island, Azores.

##### Etymology.

Named after António M. de Frias Martins, a malacologist from the University of the Azores.

##### Description.

**Figure 4.**
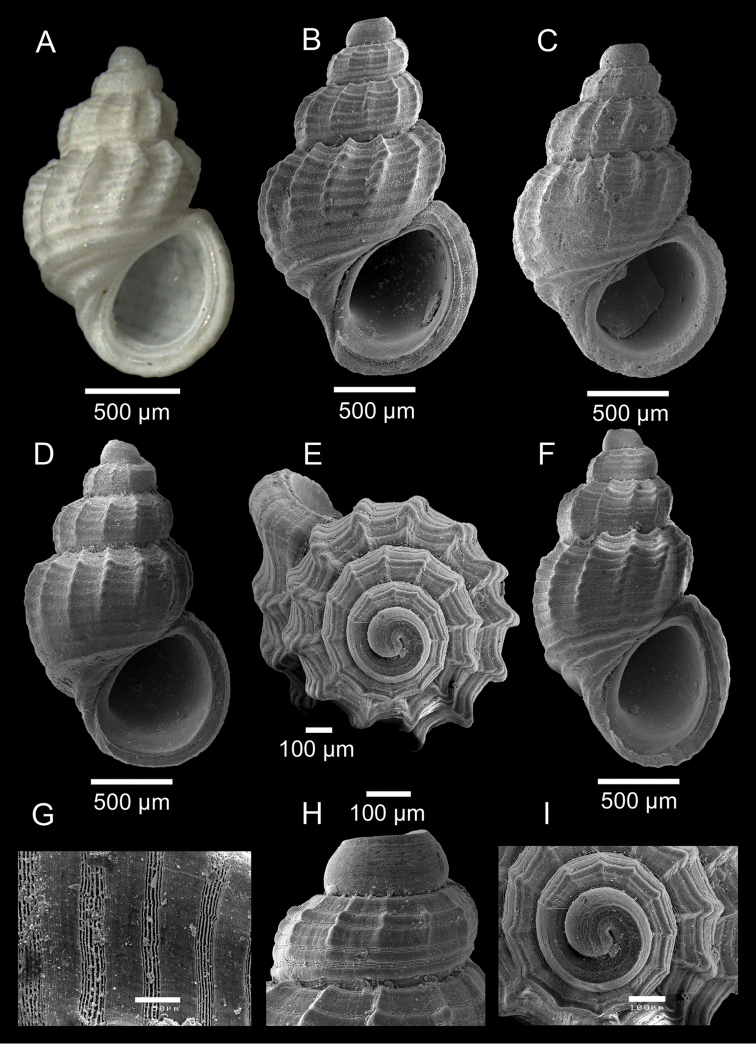
*Manzonia martinsi* sp. n. **A** Holotype DBUA 788 (1.75 × 1.13 mm) **B** Paratype 1 (1.88 × 1.20 mm), DBUA 1092 (shell) **C** Paratype 2 (1.76 × 1.14 mm), DBUA 1093 (shell) **D** Paratype 3 (1.72 × 1.10 mm), DBUA 1094 (shell) **E** Paratype 1, DBUA 1092 (shell, apical view) **F** Paratype 4 (1.76 × 1.06 mm), DBUA 1095 (shell) **G** Paratype 1, DBUA 1092 (microsculpture of the bodywhorl) **H** Paratype 1, DBUA 1092 (protoconch, lateral view) **I** Paratype 1, DBUA 1092 (protoconch, apical view).

Shell minute, white, oval-conical, up to 1.9 × 1.2 mm (Fig. 4A). Protoconch paucispiral, clearly demarcated convex whorls 1.25, diameter 340–365 µm; whorls with a pronounced keel adapically (Fig. 4I) and 5–6 faint spiral cords visible on surface between suture and keel (Fig. 4H and I). Teleoconch with 3 to 3.5 inflated, rounded, strongly convex whorls, angulated at a distance abapically from suture (Fig. 4B–F). Spire moderately high. Suture very deep, strongly constricted (Fig. 4B–F). Last whorl large, globose, 65% of shell length (Fig. 4B–D and F). Base large, rounded (Fig. 4B–D and F). Spiral sculpture consisting of flat cords (5–6 on first teleoconch whorl, 8–9 on penultimate whorl and 9–10 on body whorl) equal to two times broader than the interspaces ornamented with minute pits arranged in spiral lines (Fig. 4G); interspaces with 4 to 6 raised spiral striae (Fig. 4G). Abapical area of body whorl (base) with two strong spiral cords separated by broad spiral depression; third spiral cord located nearer the lip may be present (Fig. 4B and F). Axial sculpture consisting of 10–14 flexuous, opisthocline, moderately broad and strongly elevated ribs (body whorl), fading on base (about 7–8th spiral cord) (Fig. 4B, D and F). Intersection of axial and spiral sculptures raising small knobs (Fig. 4E). Aperture oval, strongly thickened, oblique with continuous peristome (Fig. 4B–D and F). Parietal region slightly convex (Fig. 4B–D and F). Outer lip with very thickened edge (smooth inside) (Fig. 4B–D and F). Inner lip moderately thickened and slightly concave (Fig. 4B–D and F). Umbilicus lacking (Fig. 4B–D and F).

##### Habitat.

In the infralittoral on rocks covered by algae.

##### Geographical distribution.

São Miguel Island. Probably endemic to the Azores.

##### Remarks.

*Manzonia martinsi* sp. n. is similar in shell shape to *Manzonia lusitanica* Gofas, 2007 and *Manzonia crispa* (Watson, 1873). It differs from *Manzonia lusitanica* in having a lower number of spiral cords on the body whorl (9–10 in the new species and 12–13 in *Manzonia lusitanica*). *Manzonia martinsi* sp. n. has 9–10 spiral cords on the body whorl and 4–6 raised spiral striae on the interspaces between cords, while *Manzonia crispa* has 7 spiral cords on the body whorl and 8–10 lower spiral striae between cords. *Manzonia martinsi* sp. n. differs from *Manzonia arata* Gofas, 2007 in the number of raised spiral striae on the interspaces as well as on the number of spiral lines with pits on the spiral cords, which are lower 2 to 3 times in the new species (Table 1).

**Table 1.** Comparison of the conchological characters of *Manzonia arata*, *Manzonia crispa*, *Manzonia lusitanica*, and *Manzonia martinsi* sp. n.

Species	Number of spiral cords adapical area (body whorl)	Number of spiral cords abapical area (body whorl)	Number of axial cords (body whorl)	Number of raised spiral threads (interspaces)	Number of spiral lines with pits (spiral cords)	Interspaces and spiral cords
*Manzonia arata*	8–9	3	12–13	10–12	9–10	Spiral cords narrower than the interspaces.
*Manzonia crispa*	7	4	10–12	8–10	8–10	Interspaces three times broader than spiral cords.
*Manzonia lusitanica*	12–13	2	12	3–5	3–6	Spiral cords as broad as interspaces or slightly broader.
*Manzonia martinsi* sp. n.	9–10	3	10–14	4–6	4–6	Spiral cords equal up to two times broader than interspaces.

## Discussion

### Biodiversity and endemicity

The published information on the shallow-water rissoids of the Azores was compiled by Ávila (2000, 2005) and subsequently supplemented by Ávila et al. (2012), who analysed a revised and updated checklist with the entire Atlantic and Mediterranean rissoid fauna.

This current work corrects previous misidentifications; and the novelties described herein increase the rissoid fauna of the Azores to 39 species. Twenty-nine rissoid species are known only from the shallow-waters in the Archipelago, 20 of these have also been reported from the late Pleistocene outcrops of Santa Maria Island (Marine Isotope Stage (MIS) 5e, around 120–130 kya), which corresponds to the last interglacial period: *Alvania abstersa* van der Linden & van Aartsen, 1994, *Alvania angioyi* van Aartsen, 1982, *Alvania cancellata* (da Costa, 1778), *Alvania cimicoides* (Forbes, 1844), *Alvania formicarum* Gofas, 1989, *Alvania mediolittoralis* Gofas, 1989, *Alvania poucheti* Dautzenberg, 1889, *Alvania sleursi* (Amati, 1987), *Alvania tarsodes* (Watson, 1886), *Botryphallus ovummuscae* (Gofas, 1990), *Cingula trifasciata* (J. Adams, 1800), *Crisilla postrema* (Gofas, 1990), *Manzonia unifasciata* Dautzenberg, 1889, *Merelina tesselata* (Schwartz, 1860), *Onoba moreleti* Dautzenberg, 1889, *Pusillina inconspicua* (Alder, 1844), *Rissoa guernei* Dautzenberg, 1889, Setia cf. alexandrae, *Setia subvaricosa*, and *Zebina paivensis* (Watson, 1873) (=*Zebina vitrea*) (Ávila et al. 2002, 2009a, 2009b, 2010, and present study) (Table 2).

**Table 2.** Checklist of the Rissoidae species reported from the Azores. The records of *Crisilla iunoniae* [Terceira Island (Praia da Vitória, 38°43'N, 27°04'W, sandy beach)] and *Rissoa mirabilis* [Santa Maria Island (CANCAP-V expedition, 36°59'N, 25°02'W, 35 and 55 m depth)] are based on Hoenselaar and Goud (*in litt.*, 2002); MIS 5e: Marine Isotope Stage 5e, around 120–130 kya; ?: species not confirmed; *: only fossils known (age not determined); Shallow: intertidal down to 50 m depth; Deep: below 50 m depth; Sh-De: species occurring from shallow to deep-waters.

Species	Recent	Fossil record (MIS 5e)	Bathymetric zonation	Azorean endemic
*Alvania abstersa* Van der Linden & Van Aartsen, 1994	1	1	Shallow	1
*Alvania adiaphorus* Bouchet & Warén, 1993	1		Deep	
*Alvania adinogramma* Bouchet & Warén, 1993	1		Deep	
*Alvania angioyi* Van Aartsen, 1982	1	1	Shallow	1
*Alvania cancellata* (Da Costa, 1778)	1	1	Shallow	
*Alvania cimicoides* (Forbes, 1844)	1	1	Deep	
*Alvania formicarum* Gofas, 1989	1	1	Shallow	1
*Alvania internodula* Hoenselaar & Goud, 1998	1		Shallow	1
*Alvania lamellata* Dautzenberg, 1889	1		Deep	1
*Alvania mediolittoralis* Gofas, 1989	1	1	Shallow	
*Alvania multiquadrata* van der Linden & Wagner, 1989	?	*	Shallow	
*Alvania nonsculpta* Hoenselaar & Goud, 1998	1		Deep	1
*Alvania platycephala* Dautzenberg & Fischer, 1896	1		Deep	
*Alvania poucheti* Dautzenberg, 1889	1	1	Shallow	1
*Alvania sleursi* (Amati, 1987)	1	1	Shallow	
*Alvania stenolopha* Bouchet & Warén, 1993	1		Deep	
*Alvania tarsodes* (Watson, 1886)	1	1	Shallow	1
*Alvania zoderi* Hoenselaar & Goud, 1998	1		Deep	1
*Amphirissoa cyclostomoides* Dautzenberg & Fischer, 1897	1		Deep	
*Benthonella tenella* (Jeffreys, 1869)	1		Deep	
*Benthonellania fayalensis* (Watson, 1886)	1		Deep	
*Botryphallus ovummuscae* (Gofas, 1990)	1	1	Shallow	1
*Cingula trifasciata* (J. Adams, 1800)	1	1	Shallow	
*Crisilla iunoniae* (Palazzi, 1988)	1		Shallow	
*Crisilla postrema* (Gofas, 1990)	1	1	Shallow	
*Manzonia martinsi* sp. n.	1		Shallow	1
*Manzonia unifasciata* Dautzenberg, 1889	1	1	Shallow	1
*Merelina tesselata* (Schwartz, 1860)		*	Shallow	
*Obtusella intersecta* (S.V. Wood, 1857)	1		Sh-De	
*Obtusella roseotincta* (Dautzenberg, 1889)	1		Deep	1
*Onoba moreleti* Dautzenberg, 1889	1	1	Shallow	1
*Pseudosetia azorica* Bouchet & Warén, 1993	1		Deep	
*Pusillina inconspicua* (Alder, 1844)	1	1	Sh-De	
*Rissoa guernei* Dautzenberg, 1889	1	1	Shallow	
*Rissoa mirabilis* (Manzoni, 1868)	1		Shallow	
*Setia alexandrae* sp. n.	1	1	Shallow	1
*Setia ambigua* (Brugnone, 1873)	1		Shallow	
*Setia ermelindoi* sp. n.	1		Shallow	1
*Setia netoae* sp. n.	1		Shallow	1
*Setia quisquiliarum* (Watson, 1886)	1		Shallow	1
*Setia subvaricosa* Gofas, 1990	1	1	Shallow	1
*Zebina paivensis* (Watson, 1873)		*	Shallow	
**Total**	39	20		19

Although *Obtusella intersecta* (S.V. Wood, 1857) is a deep-water species in the Azores (the shallowest record is from Faial, at 75 m depth – Hoenselaar and Goud in litt. 2002), it is also found in shallow-waters in other sites from its wide geographical range (e.g., 15–34 m depth at Mauritanian shores, CANCAP III; 22–50 m depth at Cape Verde, CANCAP VI; 20–35 m depth, Bonden, Sweden (58°12'N, 11°20'E); all data from Hoenselaar and Goud in litt. 2002) (Table 2).

Hoenselaar and Goud (in litt. 2002) reported *Alvania multiquadrata* van der Linden & Wagner, 1989 as a living species in the Azores. However, we did not find recent specimens of this taxon in the area. *Alvania multiquadrata* was only found as fossil shells by the CANCAP expeditions (CANCAP-V, Sta. 5.071, south of São Miguel Island, 37°49'N, 25°25'W, at 220 m depth, on gravel bottoms) (Table 2).

The Rissoidae is the most species-rich molluscan family in the Archipelago of the Azores. It also contains the largest number of endemic marine species in the region: 19 endemics (48.7%), if we consider all rissoids; or 15 (51.7%), if we consider only the 29 shallow-water species. All 19 Azorean endemic rissoids (15 shallow and 4 deep-water species) possess a non-planktotrophic mode of larval development.

The last account on the shallow-water marine molluscs from the Azores reports 423 taxa (Ávila, unpublished data), of which 6 are introduced species (Cardigos et al. 2006) and 34 are pelagic species. If these 40 species are discounted, there are 383 shallow-water benthic molluscs, of which 41 are endemic to the Azores (10.7% of endemisms). We note that the Rissoidae constitutes 36.6% of all endemic shallow-water benthic molluscs from the Azores, which further highlights the contribution of this family to regional biodiversity.

## Supplementary Material

XML Treatment for Setia

XML Treatment for Setia alexandrae

XML Treatment for Setia ermelindoi

XML Treatment for Setia netoae

XML Treatment for Manzonia

XML Treatment for Manzonia martinsi
